# Subjective Well-Being From a Just-World Perspective: A Multi-Dimensional Approach in a Student Sample

**DOI:** 10.3389/fpsyg.2019.01739

**Published:** 2019-07-30

**Authors:** Sofya Nartova-Bochaver, Matthias Donat, Claudia Rüprich

**Affiliations:** ^1^School of Psychology, National Research University “Higher School of Economics”, Moscow, Russia; ^2^Department of Educational Psychology, Martin Luther University of Halle-Wittenberg, Halle (Saale), Germany

**Keywords:** belief in a just world, subjective well-being, self-esteem, resilience, depressive symptoms, positive and negative affect, students

## Abstract

In a cross-sectional study with *N* = 627 individuals (*M*_age_ = 22.8, *SD*_age_ = 7.3, 147 males, 480 females, 106 non-religious, 456 religious), we investigated personal belief in a just world (BJW) as a resource for undergraduates’ subjective well-being and expected a positive relation between both constructs due to recent studies. We not only aimed at replicating but also extending recent findings by investigating a Russian sample, measuring different dimensions of well-being, and considering self-esteem and resilience as potential mediators in the relation of BJW and well-being. We also controlled for confounding effects of age, gender, religiosity, and general BJW. The findings show that personal BJW related to all investigated indicators of well-being (depressive symptoms, positive and negative affect, and mental well-being). Self-esteem mediated all relations between personal BJW and indicators of subjective well-being whereas resilience mediated relations of personal BJW to positive affect and mental well-being. The pattern of results persisted when we controlled for age, gender, religiosity, and general BJW. Our results confirm that the personal BJW world functions as a psychological resource in undergraduate students.

## Introduction

Studying at university, especially transition to university is considered one of the most important periods in the lives of intellectuals. Undergraduate students are a special group experiencing permanent everyday stress caused by high intellectual loads, competition, and assessment. Their adjustment to university is accompanied by stress, many challenges, and new experiences. In Russia, 21% of university students never graduate from it for a number of reasons (Gorbunova, 2018). Many first-year students change their life style very radically; they often separate from the parents’ home, move to another city, meet new university mates, and sometimes start their jobs (Brunsden et al., 2000; Torres and Solberg, 2001; Kuh et al., 2006; Urquhart and Pooley, 2007; Gullo et al., 2015; Bowman et al., 2018). Moreover, having coped with the adaptation of the transition to university, students still experience permanent stress caused by the preparation and passing of exams, competitions, other forms of evaluations. In addition, these difficulties which are typical for the student group are overlapping with challenges caused by the specificity of youth as a life course stage (Formica et al., 2017). Thus, being a student seems to be crucial for the development of personal identity: all these changes might require mental health and subjective well-being, and all resources to increase them are welcome. However, students may face justice/injustice in their daily lives very often, and the grades they received seem not always to be fair which might further make students anxious and vulnerable (Gorbunova, 2018).

Consequently, it seems to be especially important to investigate undergraduates’ well-being and justice-psychological factors that may strengthen it. In our study, we focused on the belief in a just world (BJW) as a possible resource for students’ well-being and expected a positive relation between both constructs due to theory and empirical findings. In fact, just-world researchers have frequently shown that the more people believed in a just world, the higher their subjective well-being level was. In more detail, a strong BJW related to better life satisfaction and less stress (Lucas et al., 2013; Khera et al., 2014), and better global well-being (Dalbert, 2002; Correia and Dalbert, 2007; Nasser et al., 2011). Furthermore, strong BJW was also connected with high endorsements of self-esteem (Dalbert, 1999; Donat et al., 2016), a decreased victimization risk in bullying situations (Kamble and Dalbert, 2012; Donat et al., 2016, 2018), a decreased stigma exposure in HIV-patients (Dorić, 2017), and positive affiliative attitudes (Sutton et al., 2017). Moreover, in line with recent research, in which subjective well-being is usually conceptualized as a multidimensional phenomenon (e.g., Diener et al., 2002), just-world researchers have investigated the relation of BJW to different dimensions of subjective well-being, for example depressive symptoms (e.g., Kamble and Dalbert, 2012) or positive and negative affect (e.g., Dzuka and Dalbert, 2002, 2007). Other studies examined well-being from a broader (e.g., Correia and Dalbert, 2007) or a multi-faceted perspective (e.g., Wu et al., 2011; Donat et al., 2016). Thus, we investigated several indicators of students’ subjective well-being in relation to their BJW. However, justice psychologists have just started to pay more attention to factors that may mediate the adaptive relation between BJW and subjective well-being, for example justice experiences in certain life domains such as teacher justice (e.g., Donat et al., 2016), and justice climate (e.g., Lucas et al., 2013). From a theoretical as well as empirical perspective, it seems plausible that self-esteem and resilience might also function as such mediators (e.g., Jiang et al., 2017).

To our knowledge, there is sparse evidence for the relation between BJW and well-being in Russia (e.g., Nartova-Bochaver et al., 2013; Astanina and Golubeva, 2014; Astanina, 2016). Thus, we focused on replicating and extending previous findings by investigating this relation in Russian undergraduates, measuring different dimensions of subjective well-being, and considering self-esteem and resilience as potential mediators in this relation.

### Belief in a Just World and Its Adaptive Functions

Belief in a just world is conceptualized as an inter-individually varying personality disposition which indicates humans’ basic need to believe that the world is a just place where everyone gets what they deserve and deserves what they get (e.g., Lerner and Simmons, 1966). This belief indicates a personal contract (Lerner, 1980) that obliges the individual to act justly who in turn expects being treated justly as well (Lerner, 2003). Due to BJW, people can handle their social environment as if it were orderly and predictable. Thus, it fulfills important adaptive functions (e.g., trust function, assimilation function; Dalbert, 2001; Dalbert and Donat, 2015). The *assimilation* function means that, facing an unjust situation, individuals try to assimilate this experience into their BJW intuitively. People can do this in many ways, for instance, by playing down the injustice, by avoiding self-focused rumination, or by forgiving. The *trust* function manifests in a person’s confidence that they are being treated justly by others (for a review, Dalbert and Donat, 2015). As a result of the assimilation and trust processes, strong just-world believers are likely to evaluate events in their own lives and events they observe as being more just and to be convinced on their good acts will be rewarded in the future. Moreover, they tend to perceive the world as an orderly and stable place which decreases their anxiety and negative feelings.

As a consequence of these adaptive functions, BJW positively related to subjective well-being in many studies. For example, individuals with a particular need to believe in a bright future defended their BJW more strongly in the face of threat (Hafer, 2000). Further, BJW negatively related to feelings of anger in an anger-evoking condition and positively with positive mood in a sadness-evoking condition (Dalbert, 2002). Hafer and Bègue (2005) reviewed additional experimental studies which provide evidence for BJW’s relation with positive and negative affect. In the same vein, questionnaire studies with students facing the school-to-work transition (e.g., Dette et al., 2004) have shown a positive association between BJW and the confidence that personal goals will be attained. Recently, Donat et al. (2016) showed that several indicators of students’ school-specific well-being significantly related to their BJW. In line with this, BJW correlated with people’s positive functioning (e.g., Dzuka and Dalbert, 2002; Fatima and Suhail, 2010; Johnston et al., 2016) and with the inhibition of negative affect in unjust situations (Strelan and Sutton, 2011). People also felt more challenged and less threatened by the need to achieve, got better results and experienced fewer negative emotions, the more strongly they endorsed the BJW (e.g., Tomaka and Blascovich, 1994). Moreover, researchers have shown that the positive association between BJW and well-being was true for various groups of victims and disadvantaged people (e.g., Otto et al., 2006; Astanina and Golubeva, 2014; Astanina, 2016) and non-victims (e.g., Otto and Schmidt, 2007).

### Subjective Well-Being

In the literature, there is a broad spectrum of concepts describing people’s positive functioning; most of them have areas of cross-sections. This functioning is indicated by subjective well-being that represents a “broad category of phenomena that includes people’s emotional responses, domain satisfactions, and global judgments of life satisfaction” (Diener et al., 1999; p. 277). More detailed, subjective well-being represents a complex construct that covers both psychological functioning and affect. It includes two distinct perspectives: the *eudaemonic perspective*, focusing on psychological functioning and self-realization, and the *hedonic perspective*, focusing on the subjective experience of life satisfaction and happiness (Ryan and Deci, 2001; Tennant et al., 2007). In this line, researchers increasingly define subjective well-being as ‘a person’s cognitive and affective evaluations of his or her life’ (Diener et al., 2002, p. 63) which is important for people’s health and development and indicates their effective coping of everyday challenges (Hascher, 2010). Nevertheless, all of these concepts reflect the subjective nature of well-being with regard to a person’s own evaluations and feelings.

The most popular markers of subjective well-being seem to be the absence of *negative affect* and the presence of *positive affect*, and *depressive symptoms*. Negative affect subsumes a broad range of negative mood states, including anxiety, fear, hostility, disgust, and scorn, and predicts subjective distress in a largest sense. In contrast, positive affect reflects people’s level of pleasurable engagement with the environment and includes enthusiasm, energy level, mental alertness, interest, joy, and determination (Watson et al., 1988; Thompson, 2007). In research on well-being, depressive symptoms are usually considered with emphasis on the affective components, such as depressed mood, but are also indicated by somatic symptoms and interpersonal problems which can occur not only in people with mental disorders but in healthy people as well (Radloff, 1977).

Sometimes researchers also use the term *mental well-being* which captures a wide conception of well-being including affective-emotional aspects, cognitive-evaluative dimensions, and psychological functioning (Tennant et al., 2007). All of these indicators demonstrate the person’s evaluation of their current personality state.

### Mediating Factors

Just-world research indicates that personal BJW and subjective well-being might be directly related or that personal BJW might even predict well-being. However, it also seems plausible that there are other psychological constructs related to personal BJW on the one hand and to subjective well-being on the other hand which might therefore mediate the direct relation of BJW to well-being. By pursuing a research design including mediating constructs, we could be able to more differentially investigate the psychological processes that regulate the relation between personal BJW and well-being Two of these constructs we thus considered in our study are self-esteem and resilience.

*Self-esteem* seems to be an important mental-health resource (e.g., Hewitt, 2009; Liu et al., 2014; Du et al., 2017; Lee et al., 2017; Mannino et al., 2017) that can help people reduce the impact of negative life events. It reflects the extent to which individuals assess themselves as being valuable, and their attitude toward themselves. Along with beliefs about oneself, self-esteem includes emotional states, such as triumph, despair, pride, and shame. In this line, it positively connected with life satisfaction and pleasant affect (e.g., Diener and Diener, 1995; Schimmack and Diener, 2003) and negatively with anxiety, depression, and unpleasant affect (e.g., Tennen and Affleck, 1993; Schimmack and Diener, 2003; Sowislo and Orth, 2013). Researchers also demonstrated a positive connection of global as well as domain-specific self-esteem with BJW (e.g., Feather, 1991; Dalbert, 1999; Donat et al., 2016) and BJW’s contribution to the maintenance of self-esteem in threatening situations, including exam stress (e.g., Dalbert, 2002; Fox et al., 2010). Consequently, we expected strong just-world believers to report a strong self-esteem which in turn can help them maintain a positive well-being. Thus, we considered self-esteem as a mediator in personal BJW’s relation to subjective well-being.

*Resilience* embodies the personal qualities that enable people to function in the face of adversity (Connor and Davidson, 2003). It reflects a person’s positive adaptation when confronted with stress or trauma (Luthar et al., 2000; Liu et al., 2014; Lee et al., 2017), an ability to keep good functioning after stress (Bonanno, 2004), and is an indicator of a successful stress-coping ability (Richardson, 2002). Hence, resilience provides a ‘buffering’ function in peoples’ lives (Cohen and Wills, 1985) and thus strongly impacts on positive mental health (Campbell-Sills and Stein, 2007). Furthermore, resilience might be positively connected with BJW due to BJW’s adaptive functions. Accordingly, several studies showed this connection in different cultural contexts (e.g., Wu et al., 2011, 2013; Riaz et al., 2015), namely China and Pakistan. To our knowledge, however, there were no surveys conducted in Western cultures yet that replicated these findings. Consequently, we expected strong just-world believers to report a strong resilience which in turn can help them maintain a positive well-being. Thus, we considered resilience as a mediator in the relation of personal BJW and subjective well-being.

### Control Factors

Studies on subjective well-being reported inconsistent results with regard to *gender* differences (in review, Diener et al., 1999, p. 292) summarized that “the magnitude of this difference was very small,” with men being slightly happier than women were. In line with these outcomes, the overall mental well-being rate was higher in men than in women (Tennant et al., 2007). In contrast, female school students reported a better subjective well-being than male school students across different well-being dimensions, as for instance worries and enjoyment (Donat et al., 2016). Other studies showed no gender differences in subjective well-being between men and women (e.g., DeNeve and Cooper, 1998). Additionally, Diener et al. (1999) discuss that men experience positive and negative aspects of subjective well-being less frequently and less strongly than women do. Because of the mixed results, gender was considered as a control factor in our study.

Another control factor tested in the current research was *age*. Evidence for a connection of subjective well-being with age is mixed (Ulloa et al., 2013). Researchers have shown that subjective well-being may remain stable across life span (Diener et al., 2006) or have various dynamics (convex, concave, linear) depending on the particular indicator and socio-demographic group. Frey and Stutzer (2002) and Deaton (2008) have revealed a convex relation between life satisfaction and age; Lang et al. (2011) have shown a decrease of prevalence and treatments of psychological distress with age. Stone et al. (2010), analyzing hedonic well-being, revealed that feelings of anger and stress declined with age, feelings of worry were increased through middle age and then declined, and feelings of sadness were in principle low. Nevertheless, most results demonstrate an increase of the subjective well-being level with age, at least from youth to old age, despite frequent dips in the middle age. Due to the inconsistent results, we included age as a control factor in the current study.

There seems to be much evidence for a favorable impact of *religiosity* on mental well-being. This connection was proven in Muslims (Ismail and Desmukh, 2012; Abdel-Khalek, 2014). A positive effect of religiosity on life satisfaction was also shown in Russian Orthodox believers (Bryukhanov and Fedotenkov, 2017). Tay et al. (2014) have shown in their meta-analysis that religiosity enhanced well-being and prevented Protestants and Catholics from depression and suicide. Hence, we considered religiosity as a control factor in our study.

Just world researchers have emphasized the necessity to differentiate between the belief in a personal just world, in which an individual is usually treated justly, and the *belief in a general just world*, in which people generally receive what they deserve (e.g., Dalbert, 1999). In accordance with the self-serving bias in fairness reasoning (Messick et al., 1985), people usually endorse the general BJW less strongly than the personal BJW. Moreover, researchers have identified different psychological functioning of both BJW dimensions in social regulation and personality: personal BJW impacts on people’s mental health and provides them with hedonic benefits, whereas general BJW provides people with the confidence of a predictable as well as ordered life and explains harsh social attitudes (for a review, see Hafer and Sutton, 2016). Thus, we focused on the personal BJW as the main predictor in our study, but also controlled for confounding effects of general BJW. Because of its adaptive properties, we expected personal BJW to be significantly related to different aspects of students’ positive functioning, that is, their subjective well-being.

### The Current Study

Based on the theory and previous findings, we tested the following hypotheses:

(1) The more people endorsed the personal BJW, the better their subjective well-being was, that is, (a) the lower the level of their depressive symptoms was, (b) the more positive affect and the less negative affect they reported, and (c) the higher the level of their mental well-being was.
(2) Self-esteem and resilience would mediate the personal BJW’s relation to well-being: we expected positive relations between personal BJW and both self-esteem and resilience which in turn would positively relate to well-being.
(3) The expected relations would be significant while controlling for people’s gender, age, religiosity, and general BJW.

To test these hypotheses, we conducted an empirical study in which we investigated the expected connections in a sample of Russian university students.

## Materials and Methods

### Sample and Procedure

In this study, *N* = 627 respondents participated; 147 were male and 480 female. Further, 456 students indicated to be religious and 106 to be non-religious, with 65 people who did not provide information about their religiosity. They were recruited from different levels (461 of them were bachelor students, 166 of them were master students) of Russian universities and represented humanities and technical specialties as follows: 421 respondents were from humanitarian and social departments (economics, literature, history, journalism, sociology, politics, psychology, philosophy, etc.); 206 respondents from natural and technical departments (math, biology, applied math, electronic engineers, etc.).

Data were collected in class; participation was scored as an optional part of their credit in the subjects “Individual Differences” and “Environmental Psychology.” “Individual Differences” were studied by bachelor students in psychology as a mandatory course; non-psychology students studied the course “Individual Differences” as minors, that is bachelor students, non-specialists in psychology, but in the same program as psychology students did. “Environmental Psychology” was an open elective course, attended by all people who were interested in this subject, that is, researchers, bachelors, master students of different specialties and external students with higher education, independent of their specialty and level.

Our sample consisted of three subsamples that differed due to the kind of well-being measurement they completed because participants were included from different studies. All respondents (*N* = 627) completed scales that measured BJW, self-esteem, resilience, and *depressive symptoms*. A subsample of *n* = 475 respondents (*M* = 24.0, *SD* = 8.0; 113 male and 362 female; 377 religious and 66 non-religious) additionally completed the scales that measured *positive and negative affect*. A subsample of *n* = 352 respondents (*M* = 22.1, *SD* = 6.5; 34 male and 318 female; 265 religious and 47 non-religious) additionally completed the scale that measured *mental well-being*.

The study was approved by the National Research University Higher School of Economics Committee on Interuniversity Surveys and Ethical Assessment of Empirical Research. All of participants have given their written informed consents.

### Measures

The *Personal Belief in a Just World Scale* (Dalbert, 1999; Russian version: Nartova-Bochaver et al., 2018) includes seven items capturing the person’s beliefs about whether they are treated fairly or not (α = 0.90; e.g., “I believe that most of the things that happen in my life are fair”). The *General Belief in a Just World Scale* (Dalbert et al., 1987; Russian version: Nartova-Bochaver et al., 2018) comprises of six items showing the extent to which a person feels the world to be just (α = 0.82; e.g., “I am confident that justice always prevails over injustice”). Responses on both BJW scales were made on a six-point scale ranging from 1 (*totally disagree*) to 6 (*totally agree*).

The 10-item *Rosenberg Self-Esteem Scale* (Rosenberg, 1965; Russian version: Prihozhan, 2002) includes statements describing how much a person values him/herself (α = 0.84; e.g., “I feel that I’m a person of worth”). Responses on this scale were made on a four-point scale ranging from 1 (*totally disagree*) to 4 (*totally agree*). The short 10-item version of the *Connor-Davidson Resilience Scale* (CD-RISC; Campbell-Sills and Stein, 2007; Russian version: Nartova-Bochaver and Astanina, 2014) comprises of statements such as “I can deal with whatever comes” (α = 0.84), each of them rated on a five-point scale ranging from 0 (*never*) to 5 (*always*).

Subjective well-being was measured using three different instruments. Firstly, we used the *Centre of Epidemiological Studies-Depression Scale* (CES-D; Radloff, 1977; Russian version: Andryushchenko et al., 2003). It includes 20 items capturing an individuals’ self-reported personal state in the past week (α = 0.88; e.g., “I felt depressed”). Two items were skipped because of very low item-total correlations (*r*_it_ = 0.06 and −0.003). Responses were made on a four-point scale ranging from 0 (*not at all or one time*) to 3 (*5 or more times per week*). Secondly, we captured positive and negative affect by using the *Positive and Negative Affects Schedule* (PANAS; Thompson, 2007; Russian version: Osin, 2012) which comprises of 10 items measuring the extent to which people felt in this way in the last week. The five items of the Positive Affect Scale (α = 0.65; e.g., “inspired”) and the five items of Negative Affect Scale (α = 0.77; e.g., “ashamed”) were rated by using a five-point scale ranging from 0 (*never*) to 4 (*extremely*). We skipped one item of the Positive Affect Scale because of a very low and negative item-total correlation (*r*_it_ = −0.09). Thirdly, we investigated mental well-being by using the *Warwick-Edinburgh Mental Well-being Scale* (WEMWBS; Tennant et al., 2007; Russian version: Robinson et al., 2013) which includes 14 statements measuring respondents’ experiences, feelings, and thoughts in the last 2 weeks (α = 0.90; e.g., “I’ve been able to make up my own mind about things”). Responses were made on a five-point scale from 1 (*none of the time*) to 5 (*all of the time*). Scale scores of all measures were formed by averaging the responses across items, reverse coding negative items as necessary.

### Analytic Strategy

The first step in our data analysis was the calculation of correlations between all investigated variables. Due to different levels of measurement, we calculated phi-coefficients between dichotomous variables (e.g., gender), point-biserial correlations between dichotomous (e.g., gender) and continuous variables (e.g., positive affect), and product-moment-correlation between continuous variables (e.g., personal BJW and positive affect).

Secondly, we used bootstrap mediation analyses and the corresponding SPSS macro developed by Preacher and Hayes (2008) to test the mediation effects. This method has various advantages over other methods (Baron and Kenny, 1986; Sobel, 1986) as it allows the inclusion of multiple covariates and is independent of normal sampling distribution. “Bootstrapping is a non-parametric resampling technique that empirically generates an approximation of the sampling distribution” (Sanches et al., 2012, p. 613). It provides users with point estimates and percentile bootstrap confidence intervals (CIs) for indirect and total effects. In accordance with Preacher and Hayes (2008) suggestion, CI were based on 5,000 bootstrap samples. To improve CI, we also used bias correction and acceleration (BCa). A CI is usually interpreted as being insignificant if it contains zero.

## Results

First, we inspected zero-order correlations between all variables (see Table 1). In accordance with Hypothesis 1a, 1b, and 1c personal BJW positively correlated with positive affect and mental well-being and negatively correlated with depressive symptoms and negative affect. The more strongly the respondents endorsed personal BJW, the more positive affect and better mental well-being they reported; the more strongly the respondents endorsed personal BJW, the less depressive symptoms and negative affect they reported. In accordance with Hypothesis 2, personal BJW was positively correlated with self-esteem and resilience. The more strongly the participants endorsed personal BJW, the stronger their self-esteem and resilience was. Self-esteem, resilience, and general BJW were also positively correlated with positive affect and mental well-being as well as negatively correlated with depressive symptoms and negative affect. Furthermore, age was related to depressive symptoms and negative affect, which in turn was more likely in men than in women. Religious participants were less likely to report depressive symptoms and negative affect and were more likely to report mental well-being than non-religious participants.

**TABLE 1:** Correlations and descriptive statistics of study variables.

	**Age**	**Gender**	**Religion**	**General BJW**	**Personal BJW**	**Self-esteem**	**Resilience**	**Depressive symptoms**	**Positive affect**	**Negative affect**	***M***	***SD***	**α**
Gender	0.10^*^												
Religion	0.11^∗∗^	0.06											
General BJW	0.05	0.11^∗∗^	0.22^∗∗∗^								3.59	0.94	0.82
Personal BJW	0.14^∗∗^	0.06	0.03	0.47^∗∗∗^							4.28	0.87	0.90
Self-esteem	0.16^∗∗∗^	–0.01	0.10^*^	0.26^∗∗∗^	0.26^∗∗∗^						3.20	0.53	0.84
Resilience	–0.20^∗∗∗^	−0.09^*^	–0.12^∗∗^	0.10^*^	0.25^∗∗∗^	0.33^∗∗∗^					2.79	0.77	0.89
Depressive symptoms	–0.16^∗∗∗^	0.06	–0.11^∗∗^	–0.21^∗∗∗^	–0.24^∗∗∗^	–0.62^∗∗∗^	–0.25^∗∗∗^				0.86	0.57	0.88
Positive affect^a^	–0.03	–0.01	–0.02	0.14^∗∗^	0.21^∗∗∗^	0.34^∗∗∗^	0.47^∗∗∗^	–0.35^∗∗∗^			2.56	0.68	0.65
Negative affect^*a*^	–0.14^∗∗^	0.10^*^	−0.11^*^	–0.13^∗∗^	–0.16^∗∗∗^	–0.39^∗∗∗^	–0.21^∗∗∗^	0.68^∗∗∗^	–0.23^∗∗∗^		1.22	0.81	0.77
Mental well-being^b^	0.08	0.04	0.19^∗∗∗^	0.33^∗∗∗^	0.42^∗∗∗^	0.63^∗∗∗^	0.38^∗∗∗^	–0.58^∗∗∗^	0.45^∗∗∗^	–0.43^∗∗∗^	3.67	0.65	0.90

For gender, 0 = male and 1 = female; for religion, 0 = not religious and 1 = religious; personal and general BJW ranged between 1 and 6, self-esteem between 1 and 4, resilience between 0 and 4, depressive symptoms between 0 and 3, positive affect and negative affect between 0 and 4, with higher values indicating a stronger endorsement of the constructs; BJW = belief in a just world; αα = Cronbach’s alpha. ^*a*^Values concerning positive and negative affect based on a subsample of *n* = 475; ^*b*^values concerning mental well-being based on a subsample of *n* = 352. Correlations: Phi-coefficient between dichotomous variables; point-biserial correlation between dichotomous and continuous variables; product-moment-correlation between continuous variables. ^*^p_*two–tailed*_ < 0.05; ^∗∗^*p*_*two–tailed*_ < 0.01; ^∗∗∗^*p*_*two–tailed*_ < 0.001.

We then tested the mediation effects of self-esteem and resilience on the relation between personal BJW and *depressive symptoms* in three models. In the first model, the mediation was tested without considering control factors which were included in the following models—demographical variables in Model 2 and general BJW additionally in Model 3 (see Table 2).

**TABLE 2:** Results from bootstrap mediation analyses for depressive symptoms (*N* = 627).

**Depressive symptoms**	**Model 1**	**Model 2**	**Model 3**
Total effect of personal BJW	−0.16^∗∗∗^ (0.03)	−0.14^∗∗∗^ (0.03)	−0.09^∗∗^ (0.03)
Direct effect of personal BJW	−0.05^*^ (0.02)	−0.04 (0.02)	−0.03 (0.03)
Direct effect of self-esteem	−0.65^∗∗∗^ (0.04)	−0.63^∗∗∗^ (0.04)	−0.62^∗∗∗^ (0.04)
Direct effect of resilience	−0.03 (0.03)	−0.05 (0.03)	−0.05 (0.03)
**Indirect effect of personal BJW through self-esteem**			
Point estimate	−0.10 (0.02)	−0.09 (0.02)	−0.06 (0.02)
95% BCa CI	[−0.14; −0.07]	[−0.12; −0.05]	[−0.10; −0.02]
**Indirect effect of personal BJW through resilience**			
Point estimate	−0.01 (0.01)	−0.01 (0.02)	−0.01 (0.01)
95% BCa CI	[−0.02; 0.01]	[−0.03; 0.002]	[−0.03; 0.002]
Partial effect of gender	—	0.09 (0.05)	0.10^*^ (0.05)
Partial effect of age	—	−0.01^*^ (0.003)	−0.01^*^ (0.003)
Partial effect of religion	—	−0.07 (0.05)	−0.06 (0.05)
Partial effect of general BJW	—	—	−0.03 (0.02)
*R*^2^	0.40	0.40	0.41

Effects represent unstandardized regression coefficients; standard errors are in parentheses. BJW, belief in a just world. For sex, 0 = male and 1 = female. For religion, 0 = non-religious and 1 = religious. BCa, bias correction and acceleration; CI, confidence interval. ^*^p < 0.05, ^∗∗^*p* < 0.01, and ^∗∗∗^*p* < 0.001.

The bootstrap results showed a significant total effect of personal BJW on depressive symptoms which decreased when self-esteem and resilience were included in the model, with the direct effect of self-esteem on depressive symptoms being significant as well. Further, there was a significant indirect effect of personal BJW on depressive symptoms through self-esteem; BCa CI for the point estimate did not contain zero. However, the effects of resilience on depressive symptoms were insignificant. The results indicate a partly mediated relation between personal BJW and depressive symptoms by self-esteem but not by resilience.

Moreover, we expected that these relations would persist when we controlled for gender, age, religiosity, and general BJW. Therefore, we repeated the bootstrap analyses while we stepwise added these variables as covariates in the model (see Table 2). After including gender (dummy-coded with 0 = male and 1 = female), age, and religiosity (dummy-coded with 0 = non-religious and 1 = religious), Model 2 showed no increased amount of explained variance. The effects of personal BJW and self-esteem on depressive symptoms were again significant. After additionally including general BJW, Model 3 showed a slightly increased amount of explained variance with significant effects of personal BJW, self-esteem, age, and gender, and an insignificant effect of general BJW. Still, the effects of resilience on depressive symptoms were insignificant. The results again indicate a partly mediated relation between personal BJW and depressive symptoms by self-esteem but not by resilience, while statistically controlling for effects of age, gender, religiosity, and general BJW, which was mainly in line with Hypothesis 3.

We then tested the mediation effects of self-esteem and resilience on the relation between personal BJW and *positive affect* as well as *negative affect* in three models each by using the same analytic strategy as before. The bootstrap results (see Table 3) showed a significant total effect of personal BJW on *positive affect* which decreased when self-esteem and resilience were included in the model; the direct effects of self-esteem and resilience on positive affect were significant as well. Moreover, the indirect effects of personal BJW on positive affect through both mediators were significant; BCa CIs for the point estimates did not contain zero. The results indicate that the relation between personal BJW and positive affect was partly mediated by self-esteem and resilience.

**TABLE 3:** Results from bootstrap mediation analyses for positive affect (*N* = 475).

**Positive affect**	**Model 1**	**Model 2**	**Model 3**
Total effect of personal BJW	0.16^∗∗∗^ (0.03)	0.17^∗∗∗^ (0.04)	0.15^∗∗∗^ (0.04)
Direct effect of personal BJW	0.07^*^ (0.02)	0.08^*^ (0.04)	0.06 (0.04)
Direct effect of self-esteem	0.20^∗∗^ (0.07)	0.24^∗∗^ (0.07)	0.23^∗∗^ (0.07)
Direct effect of resilience	0.43^∗∗∗^ (0.05)	0.43^∗∗∗^ (0.06)	0.42^∗∗∗^ (0.06)
**Indirect effect of personal BJW through self-esteem**			
Point estimate	0.02 (0.01)	0.02 (0.01)	0.02 (0.01)
95% BCa CI	[0.01; 0.05]	[0.01; 0.05]	[0.01; 0.05]
**Indirect effect of personal BJW through resilience**			
Point estimate	0.06 (0.02)	0.06 (0.02)	0.07 (0.02)
95% BCa CI	[0.03; 0.10]	[0.04; 0.10]	[0.03; 0.11]
Partial effect of gender	—	0.06 (0.07)	0.05 (0.07)
Partial effect of age	—	−0.004 (0.004)	−0.004 (0.004)
Partial effect of religion	—	−0.08 (0.08)	−0.11 (0.08)
Partial effect of general BJW	—	—	0.06 (0.04)
*R*^2^	0.25	0.24	0.25

Effects represent unstandardized regression coefficients; standard errors are in parentheses. BJW = Belief in a just world. For sex, 0 = male and 1 = female. For religion, 0 = non-religious and 1 = religious. BCa, bias correction and acceleration; CI, confidence interval. ^*^p < 0.05, ^∗∗^*p* < 0.01, and ^∗∗∗^*p* < 0.001.

Furthermore, we expected these relations to persist when controlling for gender, age, religiosity, and general BJW. Therefore, we repeated the bootstrap analyses while successively adding these variables as covariates in the model (see Table 3). After including gender, age, and religiosity, Model 2 showed no increased amount of explained variance. The effects of personal BJW, self-esteem, and resilience on positive affect were still significant. After additionally including general BJW in Model 3, the results were almost the same, with the effect of general BJW being insignificant. The results again indicate that the relation between personal BJW and positive affect was partly mediated by self-esteem and resilience while statistically controlling for effects of age, gender, religiosity, and general BJW.

The bootstrap analyses further showed a significant total effect of personal BJW on *negative affect* which decreased when self-esteem and resilience were included in the model; the direct effects of self-esteem on negative affect were also significant (see Table 4). Furthermore, the indirect effect of personal BJW on negative affect through self-esteem was significant; BCa CI for the point estimate did not contain zero. However, the effects of resilience on negative affect were insignificant. The results indicate a partly mediated relation between personal BJW and negative affect by self-esteem but not by negative affect.

**TABLE 4:** Results from bootstrap mediation analyses for negative affect (*N* = 475).

**Negative affect**	**Model 1**	**Model 2**	**Model 3**
Total effect of personal BJW	−0.15^∗∗∗^ (0.03)	−0.14^∗∗^ (0.05)	−0.11^*^ (0.05)
Direct effect of personal BJW	−0.08^*^ (0.04)	−0.08 (0.04)	−0.05 (0.05)
Direct effect of self-esteem	−0.60^∗∗∗^ (0.08)	−0.56^∗∗∗^ (0.09)	−0.55^∗∗∗^ (0.09)
Direct effect of resilience	−0.04 (0.07)	−0.06 (0.07)	−0.06 (0.07)
**Indirect effect of personal BJW through self-esteem**			
Point estimate	−0.07 (0.02)	−0.06 (0.02)	−0.05 (0.02)
95% BCa CI	[−0.11; −0.03]	[−0.10; −0.03]	[−0.90; −0.02]
**Indirect effect of personal BJW through resilience**			
Point estimate	−0.01 (0.01)	−0.01 (0.01)	−0.01 (0.01)
95% BCa CI	[−0.03; 0.01]	[−0.04; 0.10]	[−0.04; 0.10]
Partial effect of gender	—	0.21^*^ (0.09)	0.23^∗∗^ (0.09)
Partial effect of age	—	−0.01^*^ (0.01)	−0.01^*^ (0.01)
Partial effect of religion	—	−0.14 (0.10)	−0.12 (0.10)
Partial effect of general BJW	—	—	−0.05 (0.05)
*R*^2^	0.16	0.18	0.19

Effects represent unstandardized regression coefficients; standard errors are in parentheses. BJW, belief in a just world. For sex, 0 = male and 1 = female. For religion, 0 = non-religious and 1 = religious. BCa, bias correction and acceleration; CI, confidence interval. ^*^p < 0.05, ^∗∗^*p* < 0.01, and ^∗∗∗^*p* < 0.001.

Moreover, we expected that these relations would persist when we controlled for gender, age, religiosity, and general BJW. Therefore, we repeated the bootstrap analyses while we stepwise added these variables as covariates in the model (see Table 4). After including gender, age, and religiosity, Model 2 showed a slightly increased amount of explained variance. The effects of personal BJW, self-esteem, gender, and age on negative affect were significant. After additionally including general BJW in Model 3, the results were almost the same; the effect of general BJW was insignificant. Still, the effects of resilience on depressive symptoms were insignificant. The results again indicate a partly mediated relation between personal BJW and negative affect by self-esteem but not by resilience, while statistically controlling for effects of age, gender, religiosity, and general BJW.

We then tested the mediation effects of self-esteem and resilience on the relation between personal BJW and *mental well-being* in three models by using the same analytic strategy as before. The bootstrap results (see Table 5) showed that the total effect of personal BJW on mental well-being was significant. The effect decreased when we included self-esteem and resilience in the model; the direct effects of self-esteem and resilience on mental well-being were significant as well. Furthermore, the indirect effects of personal BJW on mental well-being through both mediators were significant; BCa CIs for the point estimates did not contain zero. The results indicate a partly mediated relation between personal BJW and mental well-being by self-esteem and resilience.

**TABLE 5:** Results from bootstrap mediation analyses for mental well-being (*N* = 352).

**Mental well-being**	**Model 1**	**Model 2**	**Model 3**
Total effect of personal BJW	0.34^∗∗∗^ (0.04)	0.33^∗∗∗^ (0.04)	0.26^∗∗∗^ (0.05)
Direct effect of personal BJW	0.14^∗∗∗^ (0.04)	0.13^∗∗∗^ (0.04)	0.11^∗∗^ (0.04)
Direct effect of self-esteem	0.60^∗∗∗^ (0.05)	0.57^∗∗∗^ (0.06)	0.55^∗∗∗^ (0.06)
Direct effect of resilience	0.13^∗∗∗^ (0.03)	0.18^∗∗∗^ (0.04)	0.18^∗∗∗^ (0.04)
**Indirect effect of personal BJW through self-esteem**			
Point estimate	0.16 (0.03)	0.14 (0.03)	0.10 (0.03)
95% BCa CI	[0.10; 0.21]	[0.09; 0.20]	[0.05; 0.16]
**Indirect effect of personal BJW through resilience**			
Point estimate	0.04 (0.01)	0.06 (0.02)	0.05 (0.02)
95% BCa CI	[0.02; 0.06]	[0.03; 0.09]	[0.03; 0.09]
Partial effect of gender	—	0.25^*^ (0.10)	0.25^*^ (0.11)
Partial effect of age	—	0.0001 (0.004)	0.001 (0.004)
Partial effect of religion	—	0.23^∗∗∗^ (0.07)	0.20^∗∗^ (0.07)
Partial effect of general BJW	—	—	0.05 (0.03)
*R*^2^	0.45	0.48	0.49

Effects represent unstandardized regression coefficients; standard errors are in parentheses. BJW, belief in a just world. For sex, 0 = male and 1 = female. For religion, 0 = non-religious and 1 = religious. BCa, bias correction and acceleration; CI, confidence interval. ^*^p < 0.05, ^∗∗^*p* < 0.01, and ^∗∗∗^*p* < 0.001.

Additionally, we expected these relations to persist when we controlled for gender, age, religiosity, and general BJW. Therefore, we repeated the bootstrap analyses while we stepwise added these variables as covariates in the model (see Table 5). After including gender, age, and religiosity, Model 2 showed an increased amount of explained variance. The effects of personal BJW, self-esteem, resilience, gender, and religiosity on mental well-being were significant. After additionally including general BJW in Model 3, the results were almost the same; the effect of general BJW was insignificant. The results again confirm that the relation between personal BJW and mental well-being was partly mediated by self-esteem and resilience.

## Discussion

The aim of our study was to investigate the extent to which personal BJW predicts students’ subjective well-being, how much this relation may be mediated by self-esteem and resilience, and how stable all these connections may be when controlling for age, gender, and general BJW.

According to our expectations, personal BJW related to all indicators of subjective well-being investigated in the current study. First, we found a significant negative relation of personal BJW to depressive symptoms which demonstrates that students who strongly believed their lives to be just were less often in negative mood and had less somatic disorders and interpersonal troubles than weak just-world believers. Furthermore, personal BJW was positively connected with positive affect and negatively with negative affect. These results were in line with other studies (e.g., Dzuka and Dalbert, 2007; Kamble and Dalbert, 2012; Donat et al., 2016) and also proved the adaptive functions of personal BJW, such as assimilation and trust, contributing to the more relaxed emotional state of individuals. Moreover, personal BJW correlated positively with mental well-being which is in line with all previous BJW research devoted to its connection with global well-being in students (e.g., Correia and Dalbert, 2007) as well as in other population groups (e.g., Otto et al., 2006; Wu et al., 2011). Altogether, these findings support the assumption that people’s personal BJW serves as an individual resource that strengthens their subjective well-being and helps them maintain a positive mental health which fully confirmed Hypothesis 1. Moreover, these results demonstrate that in Russia, as in other cultures investigated before, personal BJW also serves as a psychological resource through its *assimilation* and *trust* functions (Dalbert and Donat, 2015). Due to these functions, people with a high personal BJW level spend less energy on defensive behavior, and, as a result, are less anxious and healthier than weak just-world believers.

We also found indirect effects of personal BJW on these well-being indicators when we considered mediating variables. As expected, personal BJW positively correlated with self-esteem and resilience: the more strongly the participants endorsed personal BJW, the stronger their self-esteem and resilience were, which confirms results of previous studies (e.g., Dalbert, 2001; Wu et al., 2011). In accordance with previous studies (e.g., Schimmack and Diener, 2003; Campbell-Sills and Stein, 2007), self-esteem and resilience were also significantly related to all indicators of subjective well-being. Bootstrap mediation analyses further showed that the relations between personal BJW and all well-being indicators were mediated by self-esteem whereas the mediating role of resilience varied depending on the specific indicator of well-being. In detail, self-esteem partly mediated personal BJW’s effects on depressive symptoms, positive and negative affect, and mental well-being. In contrast, resilience partly mediated BJW’s effects on positive affect and mental well-being only, which represent rather positively connoted aspects of subjective well-being. However, personal BJW’s effects on depressive symptoms and negative affect were not mediated by resilience. This is somewhat surprising given that resilience was shown to be especially important for people’s adaptation or coping in stressful or traumatic situations (e.g., Luthar et al., 2000). An explanation for our results might be that we did not confront participants with such situations in our study and thus coping or adaptation was unnecessary. Nevertheless, we interpret resilience as an important mediating factor in predicting students’ maintenance of a good psychological functioning and positive feelings, although our results indicated that self-esteem seems to be even more important and might function in a more universal way. Both mediators might indicate possible psychological mechanisms through which personal BJW regulates its *trust* and *assimilation* functions.

These differential effects could be shown by investigating subjective well-being via a multidimensional approach with different indicators which also supports the theoretical conception of well-being as a broad and complex construct. Altogether, personal BJW does not only seem to directly contribute to people’s subjective well-being but also provides them with further psychological conditions that help them maintain a positive mental health such as self-esteem and resilience. Hence, Hypothesis 2 has been widely confirmed.

We further tested whether the expected relations would remain significant when controlling for confounding effects of age, gender, religiosity, and general BJW (Hypothesis 3) which was widely confirmed in our study. The correlation analyses showed that younger and female students reported more depressive symptoms and negative affect than older people and males. Moreover, females and religious people indicated that they had a better mental well-being than males and non-religious people. These results were partly in line with recent studies on the relation of subjective well-being to age (e.g., Lang et al., 2011), gender (e.g., Tennant et al., 2007), and religiosity (e.g., Bryukhanov and Fedotenkov, 2017). Further, general BJW was fully unrelated to subjective well-being which supports previous findings that personal BJW was a better predictor of subjective well-being than general BJW (e.g., Dalbert, 1999).

### Limitations

Although our work fills an important gap in the research on students’ subjective well-being and their BJW, the findings should be interpreted in light of several limitations caused mainly by the specificity of the sample. First, the sample included more female than male participants, which might have resulted in skewed findings, especially with regard to negative affect and global mental well-being. In the future, the sample should be randomized by gender. Further, our data are cross-sectional, which means that causal conclusions cannot be drawn. Longitudinal studies would allow us to evaluate the causal direction of the effects. We observed relatively weak reliability regarding our measure of positive affect. Thus, this scale might contain relatively high measurement errors. These might be due to, for example, the use of a translated version of the original instrument. Despite all accuracy in the translation process different meanings between both versions might have occurred. Thus, a more reliable Russian positive affect measure is clearly warranted. Nevertheless, the results were quite similar across all investigated dimensions of subjective well-being.

Moreover, we tested our hypotheses using bootstrap mediation analysis by Preacher and Hayes (2008). Our focus here was to investigate simple mediation effects which we derived from our theoretical, justice-psychological background. Other methods extended by Hayes (e.g., 2017) would allow the calculation of serial mediations and the inclusion of multiple moderators if needed. Further analyses such as path analyses would even allow testing multiple relations between all variables included. Future studies might thus focus on extending the complexity of model testing on the basis of an advanced theoretical background.

Although we investigated Russian university students’ subjective well-being by using a multidimensional approach, there might be other facets of well-being which also warrant consideration, such as life satisfaction. It would be interesting to investigate this dimension in relation to personal BJW in the Russian context. Due to previous studies we would expect this relation to be also positive (e.g., Correia and Dalbert, 2007). Moreover, we considered two important mediators in the relation between personal BJW and subjective well-being: self-esteem and resilience. However, both only partly mediated the expected relation which means that there might be other mediating factors, for example, justice experiences (e.g., Donat et al., 2016) which should also be addressed in future studies. Furthermore, we controlled for only one confounding psychological factor, namely general BJW, and consistently showed no significant correlations. Thus, in future studies, researchers could control for further factors to evaluate personal BJW’s effects on subjective well-being, such as neuroticism and extraversion which seem to be important predictors of well-being (e.g., Diener et al., 1999). An additional direction of developing our research would be the assessment of people’s particular ethnicity and religion in further studies as Russia is an international and multi-religious country. Finally, our study needs to be extended by investigations in more internationally diverse samples.

## Conclusion

It is obvious that more research is needed to investigate a protective role of BJW, especially of personal BJW, in the life of Russian students and students in general. As previous results gave mixed evidence concerning cultural specificity of its buffer and resource function it was very topical to scrutinize how it was in Russia. The current study showed that personal BJW related to subjective well-being and its components, such as absence of depressive symptoms and negative affect, presence of positive affect and mental well-being. In contrast, general BJW does not seem to play a significant role in stimulating subjective well-being. These findings on the one hand are in line with the most research realized in Western cultures and prove the adaptive functioning of personal BJW in Russian university students’ lives. On the other hand they may help professionals outline prospects for applications, for instance, in counseling and psychotherapy that may invent techniques how to increase personal BJW level, especially under consideration of mediating factors, such as self-esteem and resilience.

## Data Availability

The datasets generated for this study are available on request to the corresponding author.

## Ethics Statement

The study was approved by the National Research University Higher School of Economics Committee on Interuniversity Surveys and Ethical Assessment of Empirical Research. All of participants have given an informed consent. A reference can be attached by request.

## Author Contributions

SN-B has made a substantial contribution to the conception and design of the study, she was participating in drafting the work, in final approval of the version to be published; to sum up, she fully agrees to be accountable for all aspects of the work in ensuring that questions related to the accuracy or integrity of any part of the work are appropriately investigated and resolved. MD has made a substantial contribution to the main idea of the manuscript, analysis and interpretation of data, revising it critically for important intellectual content and approved finally the version to be published; and agrees to be accountable for all aspects of the work in ensuring that questions related to the accuracy or integrity of any part of the work are appropriately investigated and resolved. CR has made a substantial contribution to the analysis of data for the article and preparation of the final draft, approved finally the version to be published, and agrees to be accountable for all aspects of the work in ensuring that questions related to the accuracy or integrity of any part of the work are appropriately investigated and resolved.

## Conflict of Interest Statement

The authors declare that the research was conducted in the absence of any commercial or financial relationships that could be construed as a potential conflict of interest.
